# Acceptance of pandemic influenza A (H1N1) 2009 vaccine among health care workers, Thailand

**DOI:** 10.1186/1753-6561-5-S6-P83

**Published:** 2011-06-29

**Authors:** C Chotpitayasunondh, S Patrasuwan, M Prontri, S Poiynok

**Affiliations:** 1 Nursing, Royal Irrigation Hospital, Srinakharinwirot U., Nontaburi, Thailand

## Introduction / objectives

To identify factors associated with the acceptance of the pandemic influenza A (H1N1) 2009 vaccine among health care workers (HCWs).

## Methods

From March 1 to 31, 2009, a self-administered structured questionnaire survey was conducted among 700 HCWs to examine their knowledge, attitudes and practices toward the pandemic influenza vaccination.

## Results

The surveyed participants were composed of physician 8.5%, nurse 35%, and paramedic staff 56.5%. The response rate was 97.6%. Although 84% of the respondents regarded this vaccine as being beneficial, only 60.1% were willing to be vaccinated. The most common reason for refusal of vaccination was concern about the potential side effects of vaccine (29%). The most common source of vaccine information was broadcast media (67.8%), mainly TV and radio. Most respondents (72%) considered vaccination as the most important means for influenza control and prevention. Univariate analysis indicated that HCWs who regarded this vaccine as being safe and/or beneficial were 7 times more likely to agree to vaccinate compared to those who believed otherwise (RR: 7.08; 95% CI : 4.70-10.67). In contrast, those who had heard about vaccine adverse event from broadcast media were less likely to do so (RR: 0.77; 95% CI: 0.66-0.89). Furthermore, receiving vaccine safety information from health personnel was significantly associated with increased vaccine acceptance (RR: 2.07; 95% CI: 1.68-2.54).

## Conclusion

Effective control and prevention of disease faces a major challenge posed by the nominal level of influenza vaccine acceptance among HCWs. In order to achieve an enhanced level of influenza vaccination acceptance, the provision of up-to-date and correct vaccine information to both HCWs and the mass media is of essentiality.

## Disclosure of interest

None declared.

